# Patients in hospital with confirmed bacterial airway infection are significantly more likely to have a respiratory virus co-infection

**DOI:** 10.1099/jmm.0.001996

**Published:** 2025-07-11

**Authors:** Yunas Panikkaveettil Hamza, Mohamed Ali Ben Hadj Kacem, Naema Hassan Al Molawi, Hadi Mohamad Yassine, Hebah Atef Mohammad AlKhatib, Fatiha Benslimane, Hanan Ibrahim Kh. B. Al-Remaihi1, Reham Awni El Kahlout, Basema Ibrahim Ahmed El Kahlout, Hajar Al Khalili, Makiyeh Ahmed Al Khalili, Sanjay H. Doiphode, Emad Bashier Ibrahim Elmagboul, Javed Akhter, Einas A/Aziz Eid Al Kuwari, Peter V. Coyle

**Affiliations:** 1 Hamad Medical Corporation, Doha, Qatar; 2 Rajah Muthiah Medical College, Annamalai University, Chidambaram, India; 3 Biomedical Research Center, Member of QU Health, Qatar University, Doha, Qatar; 4 Wellcome-Wolfson Institute for Experimental Medicine, Queen's University, Belfast, UK

**Keywords:** 16S Sequencing, 16S real-time PCR, culture, dysbiosis, microbiome, pneumonia, Hospital acquired pneumonia

## Abstract

**Introduction.** Respiratory viruses are seen as cofactors in bacterial airway infection, often leading to bacterial pneumonia. This study addressed their role in hospitalized patients with bacterial infection confirmed by culture, 16S real-time PCR (16S RT-PCR) and 16S rRNA sequencing (16S Sequencing). The potential for using 16S RT-PCR and 16S Sequencing as diagnostic tools was also addressed.

**Gap Statement.** The significance of virus infections on the lung microbiome and on bacterial superinfection in hospitalized patients needs additional evidence from real-world studies.

**Aim.** The primary objective was to assess the impact of respiratory viruses on bacterial airway infection, with the secondary objective to see if 16S Sequencing had potential as a faster diagnostic tool that could augment culture.

**Methodology.** A total of 83 lower airway samples – 36 bronchoalveolar lavage fluids, 39 bronchial washes, 5 sputa and 3 endotracheal aspirates – were tested for respiratory virus and bacterial co-infection. Bacteria were tested by (a) culture, (b) 16S RT-PCR and (c) 16S Sequencing. The performance of culture-independent assays against culture was assessed, and the impact of confirmed viral infections on the airway bacterial load was determined.

**Results.** Virus infections reflected those co-circulating in the community and were significantly associated with culture and 16S Sequencing-confirmed bacterial infections [1-tailed mid P exact test (χ^2^: *P*=0.04; *P*=0.05)]. There was substantive agreement of culture and 16S RT-PCR and 16S Sequencing: kappa score: 0.66 (CI: 0.50–0.82); diagnostic accuracy 83.13% (73.32–90.46%). Virus infections were highly associated with increased bacterial load by 16S RT-PCR [2-tailed χ^2^ (χ^2^: 2.4 *P*=0.003)]. Altered microbial diversity by 16S Sequencing was seen for samples stratified by culture but not by virus detection.

**Conclusion.** Acute respiratory viral infections were significantly associated with bacterial airway infections confirmed by culture and 16S Sequencing. Airway dysbiosis was seen with bacterial-confirmed but not viral-confirmed infections, even though the latter were highly associated with increased bacterial loads using 16S RT-PCR. This suggests that virus infections induce changes in lung bacteria missed by culture and sequencing. The study supported a potential role for 16S Sequencing and 16S RT-PCR alongside culture.

## Introduction

The respiratory tract harbours communities of microbes (normal flora) in discrete niches in the nasal cavity, oropharynx and airway mucosa. Each site can vary in temperature, pH, oxygen tension and mucus production [1] and can be influenced by factors like age, gender, oral hygiene, smoking and medications [1]. Aspiration pneumonia can complicate both clinical assessment and interpretation of culture results [2], and in a hospital, the airways can harbour multidrug-resistant organisms. Defining a clinically significant bacterial load change would help assess lower respiratory tract infections (LRTIs) in hospitalized patients [3].

Viral infections, notably influenza and SARS-CoV-2, are important causes of LRTIs directly or through the expansion of the bacterial load [4]. They are also increasingly reported in hospital-acquired pneumonia (HAP) [5] and patients requiring admission to intensive care units [6]. Viral infection in this French study was confirmed in 30 out of 95 (32 %) patients, with 17 out of 95 (18 %) having virus and bacteria co-infection, the group with the highest mortality. A similar study in China, of 107 patients, showed equivalent results with virus alone in 24%, and virus–bacteria co-infection in 22% [7]. The impact of viral infections on LRTI is an important area for further work.

Bacterial culture is the gold standard for confirming LRTIs. This study looked at the association of respiratory viruses with suspected bacterial LRTI in a cohort of hospitalized patients. It also assessed the impact of viral infection on the lung bacterial load by qualitative 16S RT-PCR.

## Methods

### Study format

This was an observational longitudinal study using residual routine samples, previously tested by culture and Gram stain between November 2018 and March 2019 and reported in the validation of 16S RT-PCR in HAP [8]. The samples were also tested for respiratory viruses by RT-PCR. A total of 83 new clinical respiratory infections, involving 75 patients, were included with age and gender established from computer records. Each sample tested was from a new clinical episode.

### DNA extraction

Extraction used the EZ1 Virus Mini Kit v2.0 (QIAGEN GmbH HILDEN GERMANY) following the manufacturer’s instructions, with a sample input of 600 µl and the elution volume of 60 µl. The extracted genomic DNA (gDNA) was stored at −80 °C for subsequent processing.

### 16S RT-PCR

Primer-probe combinations (Applied TaqMan Gene Expression qPCR assays, Life Technologies Corp, Paisley, UK) were based on a previously reported pan-bacterial 16S RT-PCR assay [9]. The cycle threshold that gave the best inter-rater kappa agreement with culture, Ct≤25, was used as a threshold for positive and negative reporting [8].

### Respiratory viral RT-PCR

Samples were tested for a panel of 15 respiratory viruses using the Fast Track Diagnostics Respiratory pathogens 21 assay (FTD^™^, Luxembourg). These included the following: adenovirus (ADV); influenza A virus (IAV); influenza B virus (IAB); human rhinovirus (HRV); human coronaviruses (HCoVs) 229E, NL63, HKU1 and OC43; human parainfluenza viruses (HPIVs) 1 to 4; human metapneumovirus (HMPV); human bocavirus (HBoV); and respiratory syncytial viruses (RSVs). Community patterns of transmission were confirmed during the period using the same panel as used for the patient cohort.

### 16S Sequencing of the 16S rRNA locus

Extracted gDNA was quality-checked and made up to 10 ng per sample, and the full 16S hypervariable region (V1–V9) was sequenced using the 16S Barcoding Kit SQK-16S024 and EPI2ME Lab (EPI2ME Version 5.1.8) (Oxford Nanopore Technologies, Oxford, UK) [8]. Reads were filtered and trimmed using Trimmomatic (v0.38) to remove low-quality reads (<800 bp) and adapter sequences. Chimaeric sequences were removed, and operational taxonomic units were clustered at 95% similarity. Taxonomic assignment was carried out using the silva-based 16S classifier (release 138).

### Qualitative 16S Sequencing reporting

Specimens were confirmed as positive or negative by 16S rRNA sequencing (16S Sequencing), on the judgement of an experienced clinical microbiologist from the study. Using this heuristic approach, if one of the top 3 species in abundance was determined respectively as a respiratory pathogen or commensal bacteria, it was categorized as positive or negative. The results were assessed for performance characteristics against culture, including kappa agreement, sensitivity, specificity, positive predictive value (PPV), negative predictive value (NPV) and diagnostic accuracy.

### Microbiome diversity analysis

Out of the 83 samples, 38 passed quality filtration steps for microbiome analysis. Taxonomic classification of sequences was performed using the silva 138 classifier. Microbial diversity was estimated through alpha and beta diversity analyses using the Microbiome Analyst platform (https://www.microbiomeanalyst.ca/MicrobiomeAnalyst/).

### Alpha diversity

Microbiome alpha diversity was assessed using the Shannon diversity index to determine the change in microbial diversity between positive and negative samples stratified by (a) culture, (b) 16S Sequencing and (c) virus detection.

### Beta diversity

Microbiome beta diversity was assessed using the Bray–Curtis dissimilarity metric to determine the change in microbial diversity between positive and negative samples stratified by (a) culture, (b) 16S Sequencing and (c) virus detection.

The statistical significance of microbial community differences across the groups was tested using pairwise Permutational Multivariate Analysis of Variance (PERMANOVA). Visualization of the diversity was achieved through Principal Coordinates Analysis (PCoA).

### Statistical analysis

Descriptive statistics were presented as the median and interquartile range (IQR) for continuous variables and numbers and percentages for categorical variables. Statistical analysis was undertaken using free online statistics calculators. The median and IQR for age were calculated using BoxPlotR (http://shiny.chemgrid.org/boxplotr/). MedCalc for Windows, version 22.007 (MedCalc Software, Ostend, Belgium), was used for (a) kappa inter-rater agreement scores (https://www.medcalc.org/calc/kappa.php) and (b) sensitivity, specificity, PPV, NPV and diagnostic accuracy (https://www.medcalc.org/calc/diagnostic_test.php) between 16S Sequencing and culture; 95% CIs were reported. A 2-tailed χ^2^ and a 1-tailed mid P exact χ^2^ were used to assess the association of (a) viral detection with bacterial infection confirmed by culture and qualitative 16S Sequencing and (b) bacterial abundance by 16S RT-PCR [Epi Info^™^ 7 (CDC) (https://www.cdc.gov/epiinfo/support/downloads.html)].

## Results

### Patient cohort

The study cohort was composed of 54 male and 21 female patients aged from 21 to 93 years with a median age of 50 years (IQRs: 36.5–68 years). Patient samples included 36 bronchoalveolar lavage fluids, 39 bronchial washes, 5 sputa and 3 endotracheal aspirates (Table S1, available in the online Supplementary Material).

### Community viral transmission

**Fig. 1.**
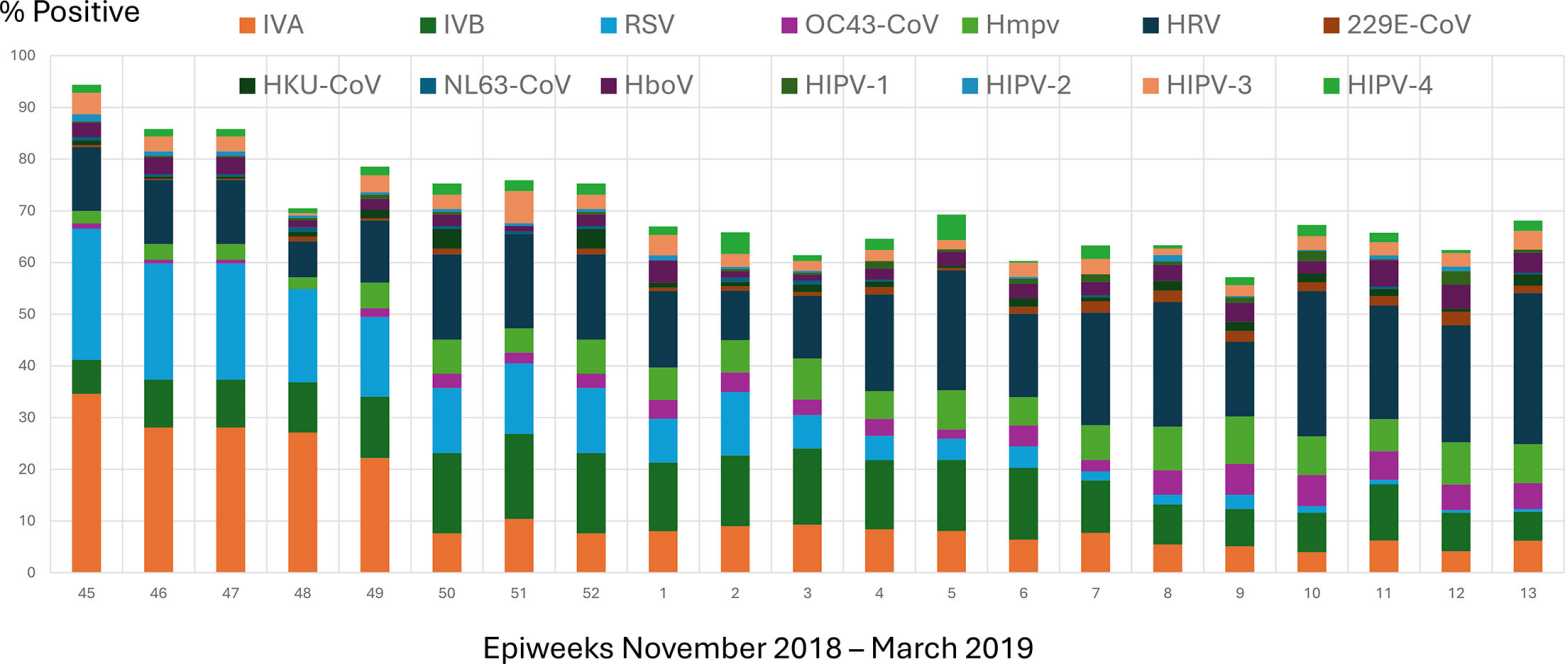
Community respiratory viruses reported in the 6-month study period. Numbers on the x-axis represent Epiweeks in 2018 and 2019, and those on the y-axis represent the % of samples positive for the respective viruses which include the following: ADV; IAV; IAB; HRV; HCoV 229E, NL63, HKU1 and OC43; HPIVs 1–4; Hmpv; HBoV; and RSV.

A total of 14 different respiratory viruses were confirmed in Qatar over the 6-month period of the study (Fig. 1). Viral waves either starting or tapering were discernible for IVA, IAB, RSV, OC43-CoV and HMPV. HRV increased in prevalence but without a discernible wave and was the commonest virus identified.

### Confirmed patient virus infections

**Fig. 2.**
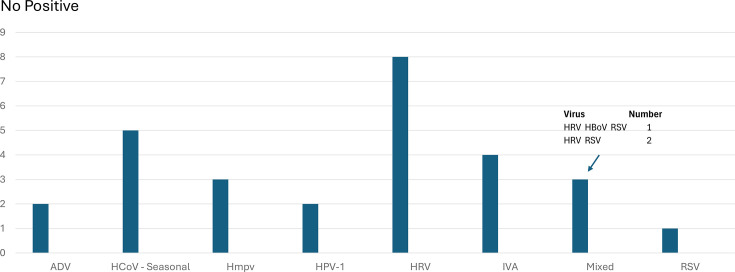
Viruses in hospitalized patients: ADV, HCoV (NL63, HKU1 and OC43), HMPV, HPIV-1, HRV, IAV, RSV and HBoV.

Viral infections were confirmed in 28 out of 83 (34%) patient episodes, with most not associated with viral waves (Fig. 2). HRV was the commonest virus seen in the hospital cohort.

### Virus association with (a) confirmed bacterial infection by culture and 16S Sequencing and (b) bacterial load disturbance by 16S RT-PCR

Table 1 shows the association of a confirmed virus infection with a bacterial infection determined by culture and 16S Sequencing. The associations respectively did not reach statistical significance with a 2-tailed χ^2^ (3.2 *P*=0.07) but did reach significance respectively with a 1-tailed mid P exact test (χ^2^: *P*=0.04; *P*=0.05). This suggests that respiratory viral infections were linked to the bacterial LRTIs. Virus infection was also highly significantly associated with an increase in the bacterial load by 16S RT-PCR [2-tailed χ^2^ (χ^2^: 2.4 *P*=0.003; 1-tailed mid P exact test χ^2^: *P*=0.003)] confirming a greater increase in the bacterial load than apparent by culture or sequencing.

**Table 1.** Viral association with bacterial infection by culture, 16S Sequencing and 16S RT-PCR

Virus status	Culture negative	Culture positive	16S Sequencing negative	16S Sequencing positive	16S RT-PCR negative	16S RT-PCR positive
Negative	33	22	30	25	30	25
Positive	11	17	10	18	6	22
2-Tailed χ^2^		χ^2^ : 3.2 *P*=0.07		χ^2^ : 2.6 *P*=0.1		χ^2^ : 8.3 *P*=0.003
1-Tailed mid P exact		χ^2^: *P*=0.04		χ^2^: *P*=0.5		χ^2^: = 0.003

The impact of respiratory virus detection on confirmation of bacterial infection determined by culture, 16S sequencing and 16S RT-PCR was assessed

RT-PCR, real-time polymerase chain reaction.

### Diagnostic performance of 16S Sequencing and 16S RT-PCR against culture

There was substantive and identical kappa score agreement and diagnostic accuracy between 16S Sequencing and 16S RT-PCR with culture: kappa score: 0.66 (CI: 0.50–0.82); diagnostic accuracy: 83.13% (73.32%–90.46%). The performance characteristics of 16S Sequencing and 16S RT-PCR against culture are shown in Table 2. 16S Sequencing had lower sensitivity (87.13%) than 16S RT-PCR (92.31%) but higher specificity, 79.55% compared to 75%.

**Table 2.** Assay performance against culture

Assay	Sensitivity (95% CI)	Specificity (95% CI)	PPV (95% CI)	NPV (95% CI)
16S RT-PCR	92.31% (79.13%–98.38%)	75% (59.66%–86.81%)	76.6% (60.06%–84.62%)	91.67% (78.54%–97.06%)
16S Sequencing	87.18% (72.57%–95.70%)	79.55% (64.70%–90.20%)	79.07% (67.57%–87.26%)	87.5% (75.29%–94.15%)

Sensitivity, specificity, PPV and NPV were assessed for 16S RT-PCR and 16S sequencing against culture

NPV, negative predictive value; PPV, positive predictive value; RT-PCR, real-time polymerase chain reaction.

### Discordant results

Discordant results were defined where culture and 16S Sequencing identified a different respiratory pathogen or where one technique failed to identify a respiratory pathogen detected by the other. There were 14 discordant results (Table S1). Of these, three samples were positive by culture and reported as scanty growth with weak mismatched Gram stains. Of the nine positive samples by 16S Sequencing, two and seven had respectively weak and negative Gram stains. The remaining two were positive by both assays but identified different bacterial species. One reported moderate growth of *Stenotrophomonas maltophilia* and a weak Gram-negative bacillus, whilst 16S Sequencing reported *Escherichia coli*. The second reported a scanty growth of *Haemophilus influenzae* with a negative Gram stain, whilst 16S Sequencing reported *Streptococcus pneumoniae*.

### Microbiome diversity in samples partitioned by virus detection and culture positivity

#### Alpha diversity

The alpha diversity analysis is reported in Table 3. There was a trend for both higher microbial richness and evenness for samples categorized as positive and negative by culture, 16S Sequencing and virus detection respectively, with 16S Sequencing and culture reaching statistical significance. This indicated a measurable change in the microbiome by each technique.

**Table 3.** Alpha diversity analysis classified by bacterial culture, 16S Sequencing and virus detection

Diversity measure	Analysis context	*P*-value	T-statistic
Shannon index	Culture	*P*=0.05	H=1.97
Shannon index	Virus	*P*=0.08	H=1.82
Shannon index	16S Sequencing	*P*=0.03	H=2.35

The Shannon Index was used as a measure of microbial diversity in populations stratified by bacterial culture, viral detection or 16S equencing.

#### Beta diversity

Table 4 shows the results of the beta diversity analysis. A highly significant difference was observed for changes in microbial diversity between samples confirmed as positive or negative by culture and 16S Sequencing. The results indicated a clustering of ~8% of the microbial community, reflecting a relative abundance shift in the samples. There was no apparent difference in the microbial communities in virus-positive and virus-negative samples.

**Table 4.** Beta diversity analysis classified by bacterial culture and virus detection

Factor	F-value	R-squared	***P*-value**
Culture	3.1654	0.080821	0.001
Virus detection	1.1718	0.031523	0.254
16S Sequencing	3.4202	0.086763	0.001

Beta Diversity was used as a measure of microbial diversity in populations stratified by bacterial culture, viral detection or 16S equencing

### Results for 16S Sequencing by PCoA

**Fig. 3.**
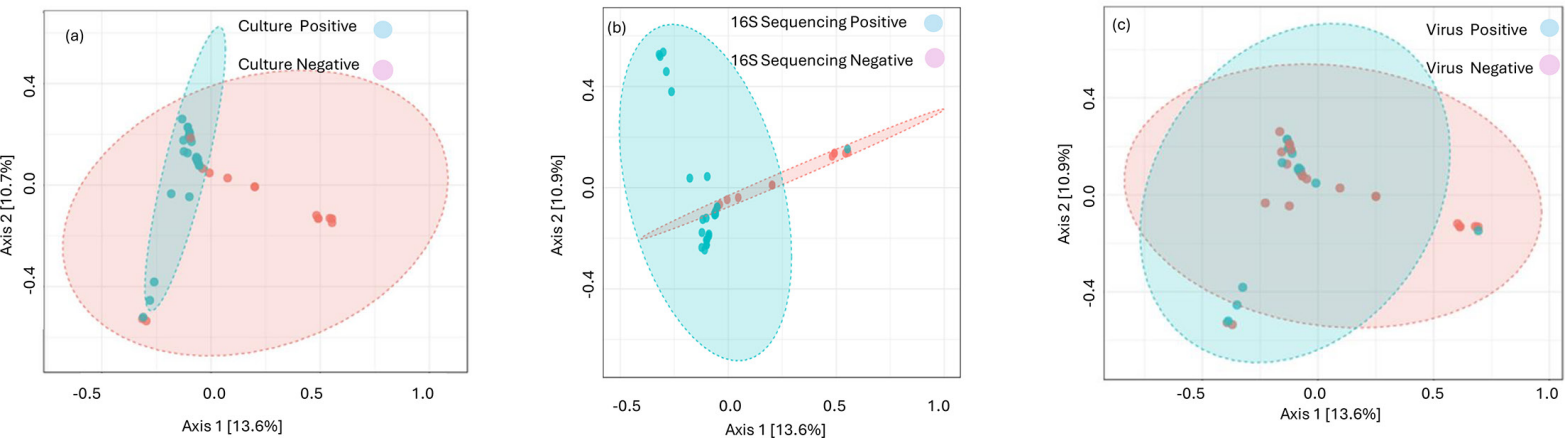
(a) Beta diversity analysis classified by bacterial detection using culture. (b) Beta diversity analysis classified by bacterial detection using 16S Sequencing. (c) Beta diversity analysis classified by virus detection.

The PERMANOVA analysis showed a significant difference in microbial composition for culture and 16S Sequencing tested samples but not when categorized by virus detection. The overlapping ellipses reflecting microbial diversity are shown in Fig. 3. The tighter ellipses suggested that the variation within these groupings was linked to altered microbial diversity (Fig. 3a, b). There was no significant separation between the microbial compositions of the virus-positive and virus-negative samples (Fig. 3c).

## Discussion

The aim of the Standards for Reporting of Diagnostic Accuracy guidelines was to improve diagnosis by reference to a gold standard [10], in this case, culture. In practice, gold standards, especially for pneumonia, show considerable disagreement, and in reality, there is no reliable gold standard to date [11], with many studies identifying no bacterial cause. For example, Jain *et al*. [12] reported a large community-acquired pneumonia study of 2,488 adults in the USA, where, in most cases, no bacterial cause was confirmed and where the commonest infections reported were respiratory viruses. The current study addressed (a) the performance characteristics of 16S Sequencing and 16S RT-PCR against culture for confirming bacterial LRTIs and (b) the impact of viral infection in a hospital setting on the diagnostic outcome and change of the lung bacterial load by 16S RT-PCR.

Taking bacterial culture as the gold standard, the performance of 16S RT-PCR and 16S Sequencing was encouraging. However, the metrics used in comparing the performance characteristics of 16S Sequencing to culture did not take account of each patient’s clinical assessment and therefore should be interpreted as providing predictive rather than diagnostic accuracy and not applicable to a real clinical setting. As expected, 16S Sequencing had higher specificity than 16S RT-PCR, respectively 79.55% (CI: 64.70%–90.20%) compared to 75% (CI: 59.66%–86.81 %), reflecting 16S RT-PCR’s inability to differentiate pathogen detection from normal flora. But the additional analysis of the microbiome raised the question of what constitutes normal flora.

Bacterial infections confirmed by culture and sequencing were significantly associated with viral infections (χ^2^; *P*≤0.05) and importantly with a change in the lung bacterial load, which showed an even greater association as determined by 16S RT-PCR (χ^2^; *P*=0.003). The confirmed bacterial infections and the increase in the bacterial load were most likely linked to mucosal inflammation [13]. The combined results of the three methods suggest that respiratory virus-induced dysbiosis may be more common in hospital LRTIs and may be missed by standard culture or sequencing. Detecting a large polymicrobial expansion of bacteria rather than a monoculture is not possible by culture, and the reporting of ‘normal flora’ may be a misnomer. Normal flora is reported when a sample is suitable for culture following a quality check by Gram stain [14] but where the culture obtained is polymicrobial. The results of this study suggest a need to reevaluate the performance of culture against a different metric, particularly for samples reporting a normal flora.

The lung microbiome communities assessed by alpha and beta diversity confirmed that those positive by culture and 16S Sequencing were significantly different from negative samples, but this was not the case for samples positive or negative by virus detection (Tables3  4, Fig. 3). The apparent normal diversity associated with confirmed virus infection is at odds with the results by 16S RT-PCR, which implied a highly significant difference. What is uncertain is whether the increase in bacterial load is real and a stepping stone to the dysbiosis seen in classically confirmed infections.

There were 14 discordant results. Of the five culture-positive samples, one and four had moderate and scanty growths reported respectively; the moderate growth had a matching weak Gram stain. The four scanty growths had two matching weak Gram stains, and two were negative. Of the 11 16S Sequencing-positive samples, 6, 3 and 2 had negative, mismatched and matched Gram stains respectively. The discordance was therefore associated with weakly positive samples, and stochastic variation linked to sampling variation due to low copy numbers may have been a contributing factor.

Against a background of many cases of pneumonia remaining culture-negative, these results suggest that molecular and sequence-based assays could augment culture. The diagnostic accuracy of 16S Sequencing reported here could help early diagnosis of pneumonia and could be reported in <12 h. Given that culture is the gold standard in this paper and the evidence for stochastic variation, the performance of 16S Sequencing is likely undervalued. A similar study using metagenomics nanopore sequencing on 33 consecutive lower respiratory tract samples from ventilated patients had 60% concordance with routine testing and detection of additional pathogens in 21% of samples [15]; the results were reportable in 7 h. The need for further research on alternative diagnostics and microbiome reporting is needed.

The bacterial flora of the respiratory tract contains many commensal bacteria capable of airway infection. In this study, there were clear limitations to the interpretation of the limited sequence coverage provided by 16S Sequencing, and without a clinical context, their clinical significance cannot be confirmed. Metagenomics could provide more certainty but currently is beyond the scope of most routine laboratories, whilst the substantive agreement shown with culture would suggest a potential role for 16S Sequencing. Laboratories considering the use of 16S Sequencing for respiratory diagnostics should consider establishing a reference list of local respiratory bacterial pathogens from their own records to help with interpretation. This study was a technical comparison of a molecular and a sequencing approach against culture for LRTIs and was not designed for assessing additional clinical parameters. Respiratory infections cover a large range of clinical presentations, and this small, single-centre study may not apply to larger multicentre studies. The balance of patients recruited was opportunistic and therefore biassed. As such, the current study cannot confirm the robustness of the observations recorded but provides unexpected findings that are worth following up in prospective studies.

## Conclusion

Community respiratory viruses were significantly associated with patients in the hospital with confirmed bacterial airway infection. In the diagnosis of LRTIs, 16S Sequencing and 16S RT-PCR had similar predictive accuracies against bacterial culture and may have a role alongside culture. An unexpected finding was a highly significant increase in the bacterial load associated with viral infection that was missed by culture and 16S Sequencing, suggesting that current diagnostics are poorly placed to confirm polymicrobial infections.

## Supplementary material

10.1099/jmm.0.001996 Table S1.

## Abbreviations

ADV: adenovirus
gDNA: genomic DNA
HAP: hospital-acquired pneumonia
HBoV: human bocavirus
HCoVs: human coronaviruses
HMPV: human metapneumovirus
HPIVs: human parainfluenza viruses
HPIV-1: human parainfluenza viruses - type 1
IAB: influenza B virus
IAV: influenza A virus
IQR: interquartile range
LRTIs: lower respiratory tract infections
NPV: negative predictive value
OTU: operational taxonomic unit
PCoA: Principal Coordinates Analysis
PERMANOVA: Permutational Multivariate Analysis of Variance
PPV: positive predictive value
RSVs: respiratory syncytial viruses
16S RT-PCR: 16S real-time PCR
16S Sequencing: 16S rRNA sequencing
